# Supplementary material for: Population-based utility scores for HPV infection and cervical squamous cell carcinoma among Australian Indigenous women
Source: PLoS One. 2021 Jul 22;16(7):e0254575. doi: 10.1371/journal.pone.0254575 (PMC8298063; doi:10.1371/journal.pone.0254575)
Supplement: S1 File — (PDF) [file pone.0254575.s001.pdf]

S1 File: Health state vignettes

Health state: Screened; cytology normal (S1)

Moira has a cervical screening test. She feels a little nervous. She also feels some discomfort while having the procedure. While waiting for her results over the next couple of weeks, Moira is a little anxious. She feels comfortable going to her local Aboriginal Community-Controlled Health Organisation and she is happy that she doesn’t have to leave behind her family or her country. She has recently lost a brother and feels worried of the impact her screening will have on other family members. However, she has a strong spiritual belief that what will be, will be and this gives her some comfort. She is able to carry out her usual activities and has no difficulty sleeping.<br>After a couple of weeks, Moira’s doctor tells her that her test results have come back normal. Her doctor recommends she comes back for a routine test in a few years.	Steps involved<br>Cervical screening test<br>↓<br>No abnormal cells<br>↓<br>Cervical screening test in few years

Health state: HPV positive with cytology normal (S2)

Agnes has a cervical screening test. Agnes feels a little nervous. She also feels some discomfort while having the procedure. While waiting for her results over the next couple of weeks, Agnes is a little anxious. However, she is able to carry out her usual activities and has no difficulty sleeping.<br>Agnes's results come back positive for HPV infection, but there are no abnormal cells. Her doctor recommends she come back in 12 months for a follow up cervical screening test.<br>Agnes feels slightly shocked and embarrassed about her HPV positive test result and is a little worried about what happens next. Her relationship with her partner is a little more difficult over the course of a couple of months.<br>Agnes is very comfortable with her doctor, who works at her ACCHO. However, she does not feel comfortable communicating with her family about her HPV status. There are some important cultural ceremonies taking place on Agnes's country, and she feels that she shouldn't take part in them. This makes her feel a little socially isolated. She is reassured that the prognosis is reasonably good, but even if it wasn't, Agnes accepts that we all have to be unwell sometimes.	Steps involved<br>Cervical screening test<br>↓<br>HPV+ve, no abnormal cells<br>↓<br>Cervical screening test in 12 months

Health state: Low grade cytology (S3)

Vera has a cervical screening test. Vera feels a little nervous. She also feels some discomfort while having the procedure. While waiting for her results over the next couple of weeks, Vera is a little anxious. However, she is able to carry out her usual activities and has no difficulty sleeping.<br>After a couple of weeks, Vera's doctor tells her that she has a an abnormality and recommends a colposcopy and biopsy. Vera is more anxious than usual and has difficulty sleeping. She is worried that she might have cancer. During the procedure there is some stinging and bleeding which lasts 3 to 4 days.<br>After a couple of weeks Vera's doctor tells her that the changes in the cell are not cancer, but a low grade abnormality. She doesn't need further treatment. Vera will need to come back to her local ACCHO in 12 months for another cervical screening test.<br>Vera had to travel to Adelaide for the colposcopy and biopsy and felt confused and ridiculed in her exchanges with hospital staff. She was not able to travel with another family member, although she did receive a travel allowance which meant she was not finacially out of pocket. She did not like being away from her country, but was grateful that she was only gone for one night. She does not feel she can share her experiences with family, but finds someone at her local ACCHO she can talk with. She is glad that her 12-month follow-up screening test with be at her local ACCHO and not require a trip to Adelaide.	<u>Steps involved</u><br>Cervical screening test<br>↓<br>Referral required<br>↓<br>Colposcopy and biopsy<br>↓<br>Confirmed low grade abnormal changes (not cancer)<br>↓<br>Cervical screening test in 12 months

Health state: High grade cytology (S4)

Gina has a cervical screening test. She feels a little nervous. She also feels some discomfort while having the procedure. While waiting for her results over the next couple of weeks, Gina is a little anxious. However, she is able to carry out her usual activities and has no difficulty sleeping.<br>After a couple of weeks, Gina's doctor tells her that she has an abnormality and recommends a colposcopy and biopsy. She is more anxious than usual and has difficulty sleeping. She is worried that she might have cancer; there has been a lot of death because of cancer in her community in the last few months. During the procedure there is some stinging and bleeding which lasts for 3 to 4 days.<br>After a week Gina's doctor tells her that the changes in the cells are not cancer but she does need to have treatment to reduce the chance of getting cancer. Even though Gina knows she does not have cancer, and accepts that being unwell happens in life sometimes, she still feels anxious and worried.<br>Gina needs treatment with a local anaesthetic. The procedure involves taking the abnormal cells out of the cervix. It is quite uncomfortable. Gina is restricted in her usual activities over the next few weeks and experiences a little bleeding in that time. She will need to come back in 4-6 months for a follow up colposcopy and cervical screening test.<br>Gina travelled with one family member to Adelaide. She experienced racism in her interactions with Adelaide-based hospital staff when undergoing her colposcopy and biopsy. This made her feel disempowered and vulnerable. Because she was not on her country, she felt that she was not able to take control of the situation. She did not want to go back to Adelaide for any more treatment, but was happy to go to her local ACCHO for a 12-month check-up	<u>Steps involved</u><br>Cervical screening test<br>↓<br>Referral required<br>↓<br>Colposcopy and biopsy<br>↓<br>Confirmed high grade abnormal changes (not cancer)<br>↓<br>Treat by removing abnormal cells

Health state Early stage cervical cancer (S5)

Allison is experiencing pain in her pelvis area, heavy discharge, bleeding between menstrual cycles and pain when she needs to go to the toilet. After a while, Allison decides to see her doctor.<br>Allison's doctor suggests that she have a colposcopy and biopsy. Allison is more worried than usual and has a bit of trouble sleeping. She is worried that she might have cancer. During the procedure there is some stinging and bleeding which lasts for 3 to 4 days.<br>After a week, the doctor tells Allison that she has early stage invasive cervical cancer. She is told that she needs to have her uterus removed to prevent the cancer from spreading. This is a major operation and Allison will need to stay in hospital for over a week. She was told that she would then start menopause, meaning that she would have no more menstrual periods, she would be unable to become pregnant and she could expect to experience hot flushes. After hearing the news, Allison becomes more anxious and worried. She has more trouble with sleeping, starts to feel unhappy and to think of herself as being worthless.<br>After being discharged from hospital, Allison sometimes experiences pain and discomfort from the procedure. She feels tired more quickly and is less able to carry out normal physical activities for a month or so. She continues to worry about whether the cancer will come back and feels unhappy on days, although she feels this way less often as time passes.<br>Allison experienced racism from some of the nurses and some of the doctors during her hospital stay. She felt angry that she didn't have the words to fight back and demand her rights. Although some of Allison's family were able to be with her, there were some important community events in her home town, so they couldn't stay with her full visit. She missed her country. The previous week two of her nieces had been removed by Families SA and placed in state care; this caused a lot of sadness and grief to Allison. Allison did not want to experience the hospital environment again, did not want to leave her family and did not want to leave her country. She made the decision that she would not have further treatment.	Steps involved<br>Symptoms<br>↓<br>Colposcopy and biopsy<br>↓<br>Early stage invasive cervical cancer<br>↓<br>Hysterectomy

Health state: Late stage cervical cancer (S6)

Celeste is experiencing pain in her pelvis area, heavy discharge, bleeding between menstrual cycles and pain when she needs to go to the toilet. After a while, Celeste decides to see her doctor.<br>Celeste's doctor suggests that she have a colposcopy and biopsy. Celeste is more worried than usual and has a bit of trouble sleeping. She is worried that she might have cancer. During the procedure there is some stinging and bleeding which lasts for 3 to 4 days after.<br>After a week, the doctor tells Celeste that she has late stage invasive cervical cancer. She is told that she needs to have chemotherapy and radiation therapy, which will occur over the course of 3-4 months and will likely have strong side effects including nausea, weight loss, loss of energy and loss of hair. She will then need to be monitored in Adelaide every three months. She was told that as a result of radiotherapy she would most likely start menopause, meaning that she would have no more menstrual periods, she would be unable to become pregnant and she could expect to experience hot flushes. After hearing the news, Celeste becomes more anxious and worried. She has more trouble with sleeping, starts to feel unhappy and to think of herself as being worthless.<br>It took four months to complete the radiation and chemotherapy, which needed to take place in Adelaide. She was able to make trips back to her community most weekends, and during the weeks was able to stay in an Aboriginal hostel provided by the hospital with some of her family. Some weeks she was on her own. She missed her children. Celeste feels tired more quickly and is less able to carry out normal physical activities for a month or so. She continues to worry about whether the cancer will come back and feels unhappy on days, although she feels this way less often as time passes.<br>Celeste experienced racism from some of the nurses and some of the doctors during her course of radiation and chemotherapy. She felt angry that she didn't have the words to fight back and demand her rights. Although some of Celeste's family were able to be with her, there were some important community events in her home town, so they couldn't stay with her full visit. She missed her country. Celeste was reluctant to engage in more chemotherapy and radiation treatment because of the impact it had on her social and emotional wellbeing, but she did understand that if the cancer did not respond well this might be the only option. She wanted this care to take place in an Aboriginal Community Controlled Health Organisation where she felt more comfortable and accepted.	<u>Steps involved</u><br>Symptoms<br>↓<br>Colposcopy and biopsy<br>↓<br>Late stage invasive cervical cancer<br>↓<br>Chemotherapy, radiation therapy<br>↓<br>Ongoing monitoring visits
