# Supplementary material for: Population-based utility scores for HPV infection and cervical squamous cell carcinoma among Australian Indigenous women
Source: PLoS One. 2021 Jul 22;16(7):e0254575. doi: 10.1371/journal.pone.0254575 (PMC8298063; doi:10.1371/journal.pone.0254575)
Supplement: Attachment — Submitted filename: Response to reviewers.docx [file pone.0254575.s002.docx]

**Responses to reviewers**

PONE-D-20-26869

Review Comments to the Author

**Reviewer #1:**

1. **Abstract: in results part should be added the statistical tests results in addition to description presentation.**

Thank you. We added the statistical tests results, such as p-values and Intra-class correlation coefficients, in the Results section (line 53-58, page 4).

2. **Methods: It should be explained why among the health utility measurement instruments; the single measurement instrument is chosen and why Standard Gamble is preferred in this study instead of other instruments**.

We added a sentence to explain why the ‘Standard Gamble’ as a single measurement instrument was chosen:

1) *‘Standard Gamble is grounded in expected utility theory, and often viewed as a gold standard to measure health utility.’* (line 113-115, page 6).
3. **Methods: What are the inclusion and exclusion criteria of the study participants?**

We added sentences to introduce the inclusion and exclusion criteria of the study participants:

1) *‘Valuations of* *the six health states were collected from 513 female Indigenous Australians aged 19+ (ranging from 19 to 78) years, residing in South Australia and taking part in a wider study examining oral HPV infection and oropharyngeal cancer in Australia.’* (line 147-150, page 8).
2) *‘Participants not enrolled during the original recruitment period will not be eligible to participate in the follow-up phases.’* (line 155-156, page 8).
