# Supplementary material for: Population-based utility scores for HPV infection and cervical squamous cell carcinoma among Australian Indigenous women
Source: PLoS One. 2021 Jul 22;16(7):e0254575. doi: 10.1371/journal.pone.0254575 (PMC8298063; doi:10.1371/journal.pone.0254575)
Supplement: Attachment — Submitted filename: Response to reviewers_R2.docx [file pone.0254575.s003.docx]

**Response to reviewers**

**Review Comments to the Author**

**Reviewer #2**: **All comments have been addressed**

Thanks.

**Reviewer #3:**

1. **I do not have the expertise to judge the specific tools used for this study however I note that the study has followed proper process for engaging with Indigenous populations, which speaks to its success overall.**

We appreciate this comment, thank you.

2. **I felt it read clearly and logically and only have minor comments/corrections:**

**1).** **Short title: Needs to include the word 'Indigenous' as that is a major focus**

We have changed short title to: ‘Utility scores for HPV infection and cervical squamous cell carcinoma among Australian Indigenous women’

**2) corrections:
(1). Line 105: take out one ‘in’ before Indigenous**

We have deleted ‘in’ (Line 105, page 6)**.**

**(2) Line 112: should the word ‘status’ actually be ‘states’ (appears twice in this line)**

Thanks. We have changed the word ‘status’ to ‘states’ (Line 113-115, page 6)

**(3) Line 113: change conduced to ‘conducted’**

We have changed the word ‘conduced’ to ‘conducted’ (Line 113, page 6)

**(4) Line 198: separates should be ‘separate’ (singular)**

We have changed the ward ‘separates’ to ‘separate’ (Line 198, page 10)

**(5) Line 282: “each health state” not states**

We have deleted ‘s’ (Line 282, page 15)

**(6) Line 328: add word to after similar- “similar to a study”**

We have added a word ‘to’ (Line 328, page 17)

**(7) Line 360: Indigenous Australian women (no ‘s’ necessary on Australian)**

We have deleted ‘s’ (Line 361, page 18)

**Reviewer #4:**

The authors have addressed a significant area of research and this article will contribute significantly towards the existing literature for cervical cancer prevention program evaluation with regards to health services uptake among indigenous communities in Australia. Yet there are some points that need to be considered:
**1) Minor confusion exists in the abstract (Methods) line 40-41. Difference is not obvious between health states 2 and 3. Whereas it has been clearly indicated between S2 (HPV positive with cytology normal) and S3 (Low grade cytology) in the results section.**

Thank you for pointing out this issue. Health state 2 (S2) should be ‘HPV-positive with cytology normal’. We have corrected it in Abstract and Methods sections (Line 41, page 3; and Line 140, page 7)

**2) The introduction section effectively gives the background of the research problem but careful proofreading would be beneficial for language errors. Similarly the discussion section has discussed the results in comparison to previous studies, yet some language errors are present.**
Thanks. We have corrected language errors:

(1). We deleted the word ‘in’ (Line 105, page 6)

(2). We added the word ‘to’ in the sentence ‘The findings are similar a study….’ (Line 329, page 17)

**3) In the data collection section, the study referenced for description of sample size (19) does not explain the participants. The references need to be rearranged correctly.**
We have rearranged references (Line 150, page 8)

**4) Statistical analytical techniques used are adequate, tables have been formed precisely and the main findings reported appropriately. However, with my limited expertise in the field of Health Economics, I am unable to advise much.**

Thanks.
